# VIM‐1 carbapenemase‐producing *Escherichia coli* in gulls from southern France

**DOI:** 10.1002/ece3.2707

**Published:** 2017-01-25

**Authors:** Marion Vittecoq, Chrislène Laurens, Lionel Brazier, Patrick Durand, Eric Elguero, Audrey Arnal, Frédéric Thomas, Salim Aberkane, Nicolas Renaud, Franck Prugnolle, Jérôme Solassol, Hélène Jean‐Pierre, Sylvain Godreuil, François Renaud

**Affiliations:** ^1^ Centre de recherche de la Tour du ValatArlesFrance; ^2^ MIVEGEC (Laboratoire Maladies Infectieuses et Vecteurs, Ecologie, Génétique, Evolution et Contrôle)UMR CNRS 5290/IRD 224Université MontpellierMontpellierFrance; ^3^ Département de Bactériologie‐VirologieCentre Hospitalier Régional Universitaire (CHRU) de MontpellierMontpellierFrance; ^4^ Université MontpellierMontpellierFrance; ^5^ INSERM U 1058Infection by HIV and by agents with mucocutaneous tropism: from pathogenesis to preventionMontpellierFrance; ^6^ Department of BiopathologyCHRUMontpellierFrance; ^7^ Department of Clinical OncoproteomicMontpellier Cancer InstituteMontpellierFrance; ^8^ UMR 5119 (UM, CNRS, IRD, IFREMER)Equipe Pathogènes et EnvironnementsU.F.R. PharmacieMontpellierFrance

**Keywords:** antimicrobial resistance, enterobacteria, humans, *Larus*, molecular characterization, phylogenetic analyses, wild birds

## Abstract

Acquired carbapenemases currently pose one of the most worrying public health threats related to antimicrobial resistance. A NDM‐1‐producing *Salmonella* Corvallis was reported in 2013 in a wild raptor. Further research was needed to understand the role of wild birds in the transmission of bacteria resistant to carbapenems. Our aim was to investigate the presence of carbapenem‐resistant *Escherichia coli* in gulls from southern France. In 2012, we collected 158 cloacal swabs samples from two gull species: yellow‐legged gulls (*Larus michahellis)* that live in close contact with humans and slender‐billed gulls (*Chroicocephalus genei*) that feed at sea. We molecularly compared the carbapenem‐resistant bacteria we isolated through culture on selective media with the carbapenem‐susceptible strains sampled from both gull species and from stool samples of humans hospitalized in the study area. The genes coding for carbapenemases were tested by multiplex PCR. We isolated 22 carbapenem‐resistant *E. coli* strains from yellow‐legged gulls while none were isolated from slender‐billed gulls. All carbapenem‐resistant isolates were positive for *bla* _VIM__‐1_ gene. VIM‐1‐producing *E. coli* were closely related to carbapenem‐susceptible strains isolated from the two gull species but also to human strains. Our results are alarming enough to make it urgently necessary to determine the contamination source of the bacteria we identified. More generally, our work highlights the need to develop more bridges between studies focusing on wildlife and humans in order to improve our knowledge of resistant bacteria transmission routes.

## 1 Introduction

Among the antimicrobial‐resistant bacteria (AMRB) of concern recently isolated from birds, a NDM‐1 carbapenem‐resistant *Salmonella* (*S. enterica* subsp. enterica serovar Corvallis) was reported in 2013 in a wild raptor (a black kite, *Milvus migrans*) in Germany (Woodford, Wareham, Guerra, & Teale, 2014).

This detection raised questions about the potential risk of the spread of resistance potentially associated with this wild reservoir. Acquired carbapenemases currently pose one of the most worrying public health threats related to antibiotic resistance (Gupta, Limbago, Patel, & Kallen, 2011; Poirel, Potron, & Nordmann, 2012). They confer resistance to carbapenems, but also to almost all β‐lactams, the most widely used class of antibiotic, and are encoded by genetic elements that are transferable between bacteria (Krahn et al., 2016; Luca et al., 2017; Potron, Poirel, & Nordmann, 2014). The actual number of carbapenemase‐producing bacterial isolates is rising, and the epidemiological status of these bacteria (sporadic versus local spread versus national endemicity) is progressively worsening worldwide (Glasner et al., 2013; WHO 2014).

Carbapenems represent the latest therapeutic innovation for β‐lactams, but this innovation is old, the latest group of molecules having been approved for clinical use more than a decade ago. Yet they are currently our last effective defense against multiresistant Gram‐negative bacteria (Woodford et al., 2014).

Our ability to limit the rise and spread of carbapenemase producers, which occur only at basal levels in many countries at present, should serve as a key performance indicator for the success or failure of the efforts that have been called for by international organizations and governments to reduce the impact of antibiotic resistance (Woodford et al., 2014). To meet this challenge, we need to investigate the role of any nonhuman reservoirs of carbapenem‐resistant bacteria, which could favor their further spread in human populations (Woolhouse, Ward, van Bunnik, & Farrar, 2015). To date, carbapenem‐resistant bacteria have been isolated from water in some rivers and sewage plants as well as in a few pets and food animals (reviewed in Woodford et al., 2014). The resistant bacteria isolated from a black kite is so far the sole evidence of the presence of carbapenem‐resistant bacteria in a wild species without any direct link with domestic animals or humans. The only other evidence of carbapenem resistance in wildlife is from a pig farm in Germany where a VIM‐1 carbapenem‐resistant *Salmonella* serovar Infantis was isolated in a mouse (*Mus musculus*) (Fischer et al., 2012). Verona integron‐encoded metallo‐β‐lactamases (VIM) belong to class B carbapenemase and were first described in Italy in 1999 (Lauretti et al., 1999). Greece is now considered to be the epicenter of the spread of VIM‐producing *Enterobacteriaceae* to other European countries where they have been subsequently detected including Spain, Italy and France (Canton et al., 2012; Mathlouthi, Al‐Bayssari, Bakour, Rolain, & Chouchani, 2016). In the light of these data, further research is clearly needed to understand the potential role of some wild species in the spread of carbapenem‐resistant bacteria.

To contribute to the development of this research, we chose to focus on *Escherichia coli* for three reasons: (1) It is a ubiquitous bacteria that can be carried by a wide range of species including humans, other mammals, and birds. (2) It is the most frequent cause of urinary tract and bloodstream infections worldwide. (3) It is a major cause of carbapenem‐resistant infection, accounting for 25% of the episodes reported in France during the last decade (INVS 2014). Hence, our aim was to investigate the presence of carbapenem‐resistant *E. coli* in a species that lives in close contact with humans following its recent colonization of urban habitats and that has subsequently experienced a strong demographic increase: the yellow‐legged gull (YLG, *Larus michahellis*; Duhem, Roche, Vidal, & Tatoni, 2008). The focal YLG population was previously reported to carry high loads of extended‐spectrum β‐lactamase (ESBL)‐producing *E. coli* (Bonnedahl et al., 2009). We also investigated the *E. coli* strains found in slender‐billed gulls (SBG, *Chroicocephalus genei*) living in the same area. We chose to study both species since they share the same environment but their feeding habits differ. YLG are opportunistic, feeding on fresh fish, but, like the black kite, they also feed on refuse and carcasses, whereas SBG mainly feed on marine fishes (Flitti, Kabouche, Kayser, & Olioso, 2009). We investigated *E. coli* carried by chicks since, within the colonies we studied, they had no contact with humans and they could only be contaminated by bacteria brought by adults or already present in the colony. Thus, finding AMRB in those chicks would mean either that these bacteria have been transmitted from adults to chicks or that the environment (surrounding water or soil) is contaminated by them.

In France, as in most Western European countries, carbapenemase‐producing bacteria infections have so far been limited to hospital settings and represent only a few cases per year. Yet the number of those cases has significantly increased in recent years in many European countries, including France (ECDC 2013; INVS 2014). In all, 913 infectious episodes associated with carbapenem‐resistant enterobacteria were reported nationwide from January 2004 to March 2014, 25% of which were due to *E. coli* strains (INVS 2014). Through the investigation of *E. coli* strains carried by gull chicks in two colonies in south‐eastern France, our aim was to elucidate four questions: (1) Are the focus populations harboring some carbapenem‐resistant bacteria while those bacteria are still rare in the neighboring human population? (2) Is the proportion of individuals carrying these AMRB similar in the two target species, despite their contrasting feeding habits? (3) Are the *E. coli* genotypes carried by gulls closely related to those recently isolated in humans in the study region? (4) Which resistance mechanisms are involved in the potentially detected resistances?

## 2 Materials and Methods

### 2.1 Ethics statement

The study has been approved by the Scientific and Ethical Council of the Tour du Valat Foundation, which is in charge of the ethical issues in our research center, on 5 October 2011. The results have subsequently been reviewed by this council. Birds were handled and sampled under the supervision of two registered bird ringers of the “Museum National d'Histoire Naturelle” of Paris (Thomas Blanchon & Yves Kayser) who made every effort to avoid any animal suffering. Permits for fieldwork were issued by the municipality of Port‐Saint‐Louis and the Communauté d'Agglomération Toulon Provence Méditerranée.

### 2.2 Sampling, bacterial strains and antibiotic susceptibility testing

Cloacal swabs were collected from gull chicks, aged from 1 to 4 weeks, in two colonies. The yellow‐legged gull colony was situated on an islet near the village of Port‐Saint‐Louis (4°51′26.50″E. 43°22′39.93″N), where 93 swabs were sampled on chicks on 23 May 2012. The colony of slender‐billed gulls was located in the Giens Peninsula (6°08′20.10″E. 43°03′01.14 N.). Sixty‐five samples were collected there on 23 July 2012.

Immediately after sampling, the swabs collected from gulls were placed in Oxoid Tryptone Soya Broth (Thermo Scientific^™^). They were then transported to the laboratory and incubated at 37°C overnight. Following incubation, a loopful was streaked on plates of Hektoen (bioMérieux, Marcy‐l'Etoile, France) and of a selective chromogenic medium for the detection of carbapenem‐resistant bacteria (Oxoid Brilliance CRE; Thermo Scientific^™^). The bacteria were incubated for 24–48 h on these media depending on observation of growth of bacterial colonies. *E. coli* identity was confirmed by matrix‐assisted laser desorption ionization–time of flight (MALDI‐TOF) mass spectrometry (Bruker Daltonik, Bremen, Germany). Antimicrobial susceptibility testing was performed by disk diffusion assay on Mueller–Hinton agar (Bio‐Rad, Marne‐la‐coquette, France) and interpreted according to the European Committee on Antimicrobial Susceptibility Testing (EUCAST 2012, version 2.0) clinical breakpoints (http://www.eucast.org/clinical_breakpoints/). Antibiotic agents tested were: amoxicillin, amoxicillin–clavulanic acid, ticarcillin, ticarcillin–clavulanic acid, piperacillin, piperacillin–tazobactam, imipenem, cephalothin, cefoxitin, cefotaxime, ceftazidime, cefpodoxime, cefpirome, cefepime, moxalactam, aztreonam. Metallo‐β‐lactamase production was investigated in *E. coli* isolated from the selective medium by the Carbapenemase/Metallo‐β‐Lactamase Confirmation Identification Pack (Rosco Diagnostic Neo‐Sensitabs^™^, Eurobio, Courtaboeuf, France).

Twenty‐five clinical isolates of carbapenem‐susceptible nonpathogenic *E. coli,* recovered from human patient's stools in the same geographical area (Montpellier hospital) and during the study period, were used for phylogenetic comparison.

In addition, four *E. coli* strains belonging to reference phylogroups (A1, B1, B2_3_, D1) were kindly provided by *Prof. Richard Bonnet*.

### 2.3 Detection and identification of carbapenem‐resistant genes

The *E. coli* strains that were isolated from the media containing carbapenem were further analyzed to determine the mechanisms involved in resistance to carbapenems using a multiplex PCR (Dallenne, Costa, Decré, Favier, & Arlet, 2010; Hornsey, Phee, & Wareham, 2011). Briefly, the presence of the most prevalent carbapenemase genes (including *bla*_KPC_, *bla*_VIM_, *bla*_OXA‐48_ group, and *bla*_IMP_) was assessed by multiplex PCR, as previously described (Dallenne et al., 2010) and *bla*_NDM_ gene presence was investigated by PCR assay (Hornsey et al., 2011). Metallo‐β‐lactamase production was assessed by the Carbapenemase/Metallo‐β‐Lactamase Confirmation Identification Pack (Rosco Diagnostic Neo‐Sensitabs^™^, Eurobio, Courtaboeuf, France).

All amplified PCR products were purified using the ExoSap purification kit (ExoSap‐it, GE Healthcare, Piscataway, NJ, USA), and bidirectional sequencing was performed using the BigDye Terminator version 3.1 Cycle Sequencing Kit (Applied Biosystems, Foster City, CA, USA) and an Applied Biosystems 3730 XL capillary sequencer. Each sequence was then compared with already known carbapenemase genes available in the NCBI database using the BLAST program. The detection of *bla*_VIM‐1_ gene was confirmed by simplex PCR assay and sequencing using the following primers: VIM_F (5′‐AGTGGTGAGTATCCGACAG‐3′) and VIM_R (5′‐TGCAACTTCATGTTATGCCG‐3′).

### 2.4 Molecular analyses

To determine the relatedness between the *E. coli* isolates sampled from YLG, SBG, and humans, we performed PCR phylotyping, discriminant single nucleotide polymorphism (SNP), multi locus sequence typing (MLST) and polymorphic variable number of tandem repeats loci (VNTR).

All isolates were assigned to the eight *E. coli* phylogenetic groups and subgroups (A0, A1, B1, A1xB1, B2_2_, B2_3_, D1, and D2) using PCR on the basis of the presence or absence of three DNA fragments (*chuA*,* yjaA*, and *tspE4.C2*) (Clermont, Bonacorsi, & Bingen, 2000) and on the comparison with four reference strains (A1, B1, B2_3_, D1).

Allele‐specific real‐time PCR was used to screen seven SNPs (*fadD234, clpX267, uidA138, clpX177, clpx234, lysP198, icdA177*) on seven housekeeping genes (Sheludchenko, Huygens, & Hargreaves, 2010) using an Applied Biosystems 7300.

Multi locus sequence typing procedures were performed using Wirth et al. MLST scheme (Wirth et al., 2006). Seven housekeeping genes (*Adk, FumC, GyrB, Icd, Mdh, PurA, RecA*) were amplified by PCR and sequenced by Eurofins (Germany). The sequences were assembled and trimmed (3,423 nucleotides in total) using the MLST Databases at the University of Warwick (freely available at http://mlst.warwick.ac.uk/mlst/). Alleles and sequence type (ST) numbers were assigned de novo in BioNumerics (Applied Maths, Sint‐Martens‐Latem, Belgium).

The genotyping assay of VNTR was performed on six polymorphic loci (CVN15, CVN7, CVN4, CVN1, CVN2, CVN14) (Lindstedt, Brandal, Aas, Vardund, & Kapperud, 2007) using a genetic analyser Applied Biosystems 7330*xl*.

### 2.5 Phylogenetic and statistical analyses

Exact Fisher tests were used to test for differences in *E. coli* phylogroup distributions among host populations.

The multiple alignments of all complete MLST sequences were conducted using ClustalW in BioEdit version 7.0.9.0. software. Maximum likelihood (ML) tree construction was based on the MLST sequences and the best‐fitting ML model under the Akaike information criterion was GTR (general time reversible) + Γ (gamma distribution) for nucleotides as identified by ModelTest (Posada & Crandall, 1998). The most likely DNA tree and corresponding bootstrap support values were obtained by PhyML using Mega 5.0 software (Tamura et al., 2011) with nearest‐neighbor interchange branch swapping and 100 bootstrap replicates. All positions containing gaps and missing data were eliminated.

Genetic polymorphism of VNTR data was measured in terms of allelic richness per locus and sample (Rs) (Mousadik & Petit, 1996), gene diversity was measured in terms of expected heterozygosity per locus and sample (Hs) using the unbiased estimator adapted to haploid data (Nei & Chesser, 1983), and the genotypic linkage disequilibrium was tested by the log‐likelihood ratio G‐statistic after Bonferroni's correction using FSTAT version 2.9.4 software (Goudet, 2003).

Multi locus sequence typing, single nucleotide polymorphism, and variable number of tandem repeats loci data were analyzed with BioNumerics software version 7.0 (Applied Maths, Sint‐Martens‐Latem, Belgium). Based on allelic profiles, the evolutionary relationship between isolates was assessed by a minimal spanning tree (MST) implemented in BioNumerics. The MST is a graphical tool that links the nodes by unique minimal paths in a given dataset: the total summed distance of all branches is minimized. The Prim's algorithm calculated a standard MST with single‐ and double‐locus variance priority rules.

## 3 Results

### 3.1 Detection of carbapenem‐resistant *Escherichia coli*

Twenty‐two *E. coli* strains isolated from yellow‐legged gulls (Table 1) were resistant to most of the β‐lactam antibiotics, except aztreonam, and were metallo‐β‐lactamase producers. Those carbapenemase‐producing *E. coli* originated from 18 of the 93 YLG chicks on which we sampled cloacal swabs. Two birds carried two carbapenemase‐producing isolates and one gull carried three. All 22 carbapenemase‐producing *E. coli* isolates were positive for *bla*_VIM‐1_ gene. Conversely, no carbapenem‐resistant bacteria were isolated from the samples collected on 65 slender‐billed gulls. Table 1 indicates the number of isolates sampled from each host group that were used for each type of analysis (Phylogroup, SNP, MLST and VNTR).

**Table 1:** Summary of the strains studied and the analyses in which they were included

Host species	Resistant to carbapenem	Number of strains	Strains for which complete data are available			
			Phylogroup	MLST	SNP	VNTR
Yellow‐legged gulls	Yes	22	22	22	22	20
	No	26	26	26	26	25
Slender‐billed gulls	No	15	15	15	15	14
Humans	No	25	25	25	25	18
Phylogroup reference strains (humans)	No	4	4	4	4	2
Total		92	92	92	92	79

### 3.2 Phylogroups

Table 2 shows the prevalence of phylogenetic groups for the entire set of isolates from the different host samples. Seven phylogroups were determined in susceptible *E. coli* samples and only three phylogroups (A1, A1xB1, B2_2_) in VIM‐1‐resistant *E. coli* isolates. The distribution of phylogroups in resistant isolates was significantly different from that observed in susceptible samples (*p < *.001 Fisher's exact test). Among the 22 carbapenem‐resistant isolates, 18 belonged to the phylogroup A1 (81.8%). This proportion was significantly higher than that found in the susceptible isolates as a whole (*p < *.001 Fisher's exact test).

**Table 2:** Prevalence of eight *Escherichia coli* phylogroups in the strains isolated from yellow‐legged gulls, slender‐billed gulls, and human patients

Sample group	*n*	Number of strains of each phylogenetic groups (% in the sample group)							
		A0	A1	A1 × B1	B1	B2_2_	B2_3_	D1	D2
Resistant *E. coli* from yellow‐legged gulls	22	0	18 (81.8%)	2 (9.1%)	0	2 (9.1%)	0	0	0
Susceptible *E. coli* from yellow‐legged gulls	26	6 (23.1%)	4 (15.4%)	2 (7.7%)	10 (38.4%)	0	0	4 (15.4%)	0
Susceptible *E. coli* from slender‐billed gulls	15	1 (6.7%)	1 (6.7%)	0	6 (40.0%)	0	0	4 (26.6%)	3 (20.0%)
Susceptible *E. coli* from humans	25	4 (16.0%)	2 (8.0%)	0	6 (24.0%)	0	7 (28.0%)	4 (16.0%)	2 (8.0%)

### 3.3 Multilocus sequence typing

**Figure 1:**
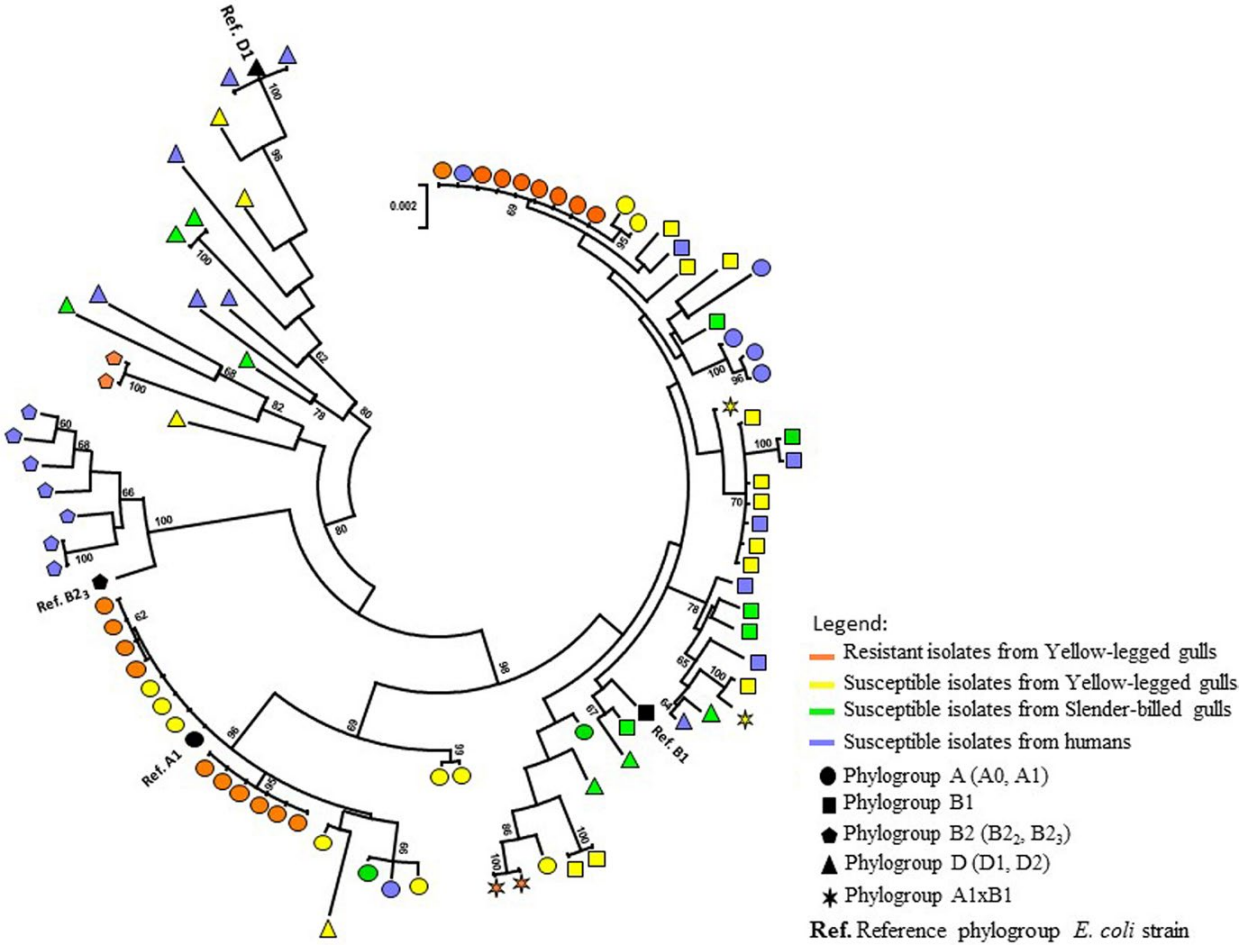
Phylogenetic relationships among the 92 *Escherichia coli* isolates studied based on concatened MLST sequences. The circle tree was constructed using maximum likelihood methods. Bootstrap values greater than or equal to 60% are indicated at the nodes, and those relating to the five clusters containing carbapenem‐resistant isolates are shown in blue. The *E. coli* strains were isolated from yellow‐legged gulls (YLG), slender‐billed gulls (SBG), and humans

The MLST analysis involved 92 nucleotide sequences (Table 1) with 7 partial housekeeping gene sequences (i.e., 4,963 nucleotides in total). Among the 360 variable sites, 255 were informative sites over the 4,963 bases. The number of unique haplotypes was 68 out of 88 in total, excluding the four reference strains. The PhyML tree (Figure 1) with bootstraps based on concatenated 7 fragments of sequences which totalized 4,963 nucleotides showed that the resistant isolates (orange symbols) were grouped in five clusters grouping 4 (phylogroup A), 6 (phylogroup A), 2 (phylogroup AxB), 8 (phylogroup A), and 2 (phylogroup B) isolates. We noticed that these five clusters were scattered in the phylogenetic tree (Figure 1) with blue node bootstrap of 62, 95, 86, 69, and 82, respectively. The genetic diversity is more extended in susceptible strains due to the number of clusters grouping together.

### 3.4 Single nucleotide polymorphism

In the SNP analysis, 26 distinct haplotypes were detected among the 88 *E. coli* isolates studied (reference strains were not included, see Table 1). The number of SNP haplotype combinations was as follows: 3 SNP haplotypes out of 22 isolates in resistant *E. coli* from yellow‐legged gulls, 11 out of 26 isolates in susceptible *E. coli* from yellow‐legged gulls, 11 out of 15 isolates in susceptible *E. coli* from slender‐billed gulls, and 14 out of 25 isolates in susceptible *E. coli* from humans. The diversity of SNP found in the carbapenem‐resistant isolates (3 out of 27 haplotype combinations) was lower than in other strains, but this difference was not significant (*p* = .07 Fisher's exact test). Elsewhere, the CCCGCCT (*fadD234, clpX267, uidA138, clpX177, clpx234, lysP198, icdA177*) SNP combination was significantly more frequent in VIM‐1 strains (18 out of 22) than the others (*p *< .001, Fishers's exact test) and absent in the *E. coli* isolates sampled in slender‐billed gulls (see SNP details in Supplementary Information—Table S1).

### 3.5 Variable number tandem repeat

The VNTR gave complete results for 79 isolates only (Table 1). All the loci were polymorphic, showing from 3 (CVN7 and CVN15) to 14 alleles (CVN14). There was lower genetic variability in the sample of resistant isolates as shown by the allelic richness (*R*s = 2.3 ± 1.2) vs. other samples (susceptible isolates from yellow‐legged gulls *R*s = 4.5 ± 3.6; susceptible isolates from slender‐billed gulls *R*s = 3.0 ± 2.2; susceptible isolates from humans *R*s = 4.3 ± 2.9). However, the difference of Rs between each pair of samples was not significant (Wilcoxon test, *p*‐values range between .574 and .936).

Elsewhere, the gene diversities per locus were similar in all samples except for locus CVN2 which showed a higher value for resistant YLG strains (Hs = 0.69) compared to the three other sample groups (susceptible strains from YLG: Hs = 0.22, SBG: Hs = 0.26, humans: Hs = 0.17). However, the difference of Hs between each pair of samples was not significant (Wilcoxon test, *p*‐values range between .261 and .936).

A significant linkage disequilibrium (*p* < .001) was underscored at 3 loci pairs: (CVN1 × CVN2, CVN1 × CVN14, CVN2 × CVN14) in the resistant YLG sample group, whereas no significant linkage disequilibrium was detected in any loci combination in other samples, even in the susceptible *E. coli* isolated from YLG.

### 3.6 Phylogenetic analysis based on MLST, SNP, and VNTR

**Figure 2:**
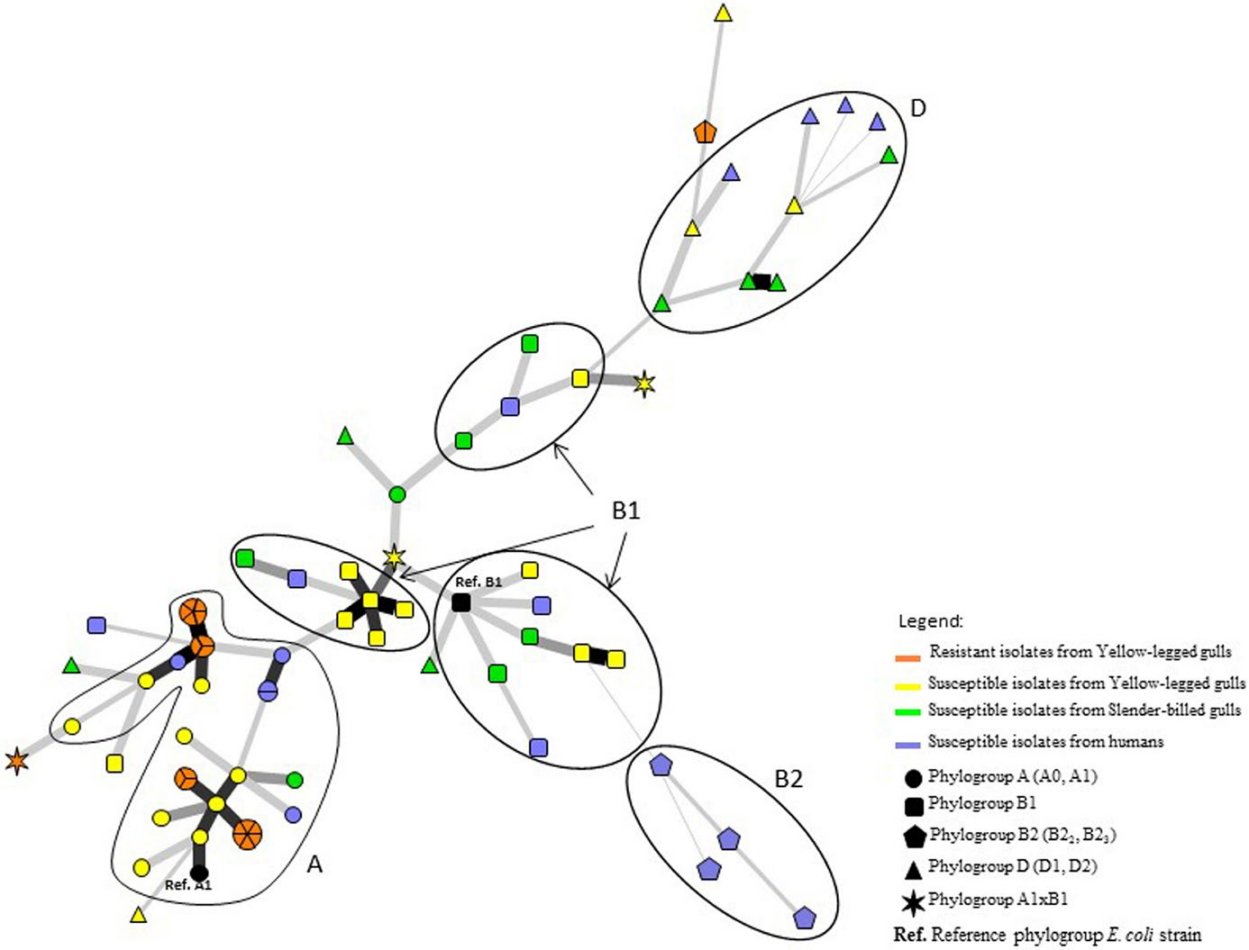
Minimum spanning tree of 79 of the studied *Escherichia coli* strains based on MLST, SNPs and VNTR. The 13 strains for which part of the VNTR data was missing were excluded. The phylogroups are shown as ovals. Clonal complexes are indicated by symbols proportional in size to the number of strains within them. Black lines connecting strains indicate that they differ at (1) least by one VNTR (bold thick lines) to two VNTR and (2) seven markers (five MLST genes and two SNPs (the thinnest lines))

The minimum spanning tree presented in Figure 2 is based on genetic sequence similarity according to MLST (7 fragments of gene sequences shortened and aligned to the reference sequences in the MLST Databases at the University of Warwick (freely available at: http://mlst.warwick.ac.uk/mlst/) (i.e., 3,423 nucleotides in total)), SNP and VNTR analyses. The phylogenetic structure highlighted is similar to that underlined in Figure 1. Isolates of the same phylogroup tend to cluster together. Carbapenem‐resistant isolates do not form a distinct cluster. Conversely, they are grouped in three clusters that also include carbapenem‐susceptible strains isolated from the different host species (YLG, SBG, and humans). In addition, only one resistant isolate presents a unique sequence, the others are grouped in five clonal complexes including 2–6 strains.

In order to test for potential artifacts in the deep branches caused by VNTR which tend to have a very rapid dynamics, we led the same analysis without VNTR, i.e., MLST + SNP. The results obtained show the same network topography; the only minor difference concerns the B2 group position, but all strains of this group cluster together as observed in Figure 2 (see Supplementary Information—Figure S1).

## 4 Discussion

We highlighted the presence of VIM‐1 carbapenem‐resistant *E. coli* strains in yellow‐legged gulls in southern France. Our results confirm that gulls represent a bird group that frequently carries antimicrobial‐resistant bacteria, as was previously shown in several studies (e.g., Čížek, Dolejská, Karpíšková, Dědičová, & Literák, 2007; Hasan, Melhus, Sandegren, Alam, & Olsen, 2014; Poirel, Potron, De La Cuesta et al., 2012) led in particular in southern Europe (Stedt et al., 2014).

Alarmingly, while the previous report of carbapenem‐resistant bacteria in a wild bird was a single case in a raptor (Fischer, Schmoger, Jahn, Helmuth, & Guerra, 2013), we detected VIM‐1‐bearing *E. coli* carriage in 18 different chicks, which raises the question of the extent of wildlife contamination in the study region. Interestingly, we identified 5 clonal complexes and one unique genotype within the VIM‐1‐containing bacteria we detected (Figure 2). This suggests that several distinct introductions of carbapenem‐resistant *E. coli* occurred on the islet. Further studies are needed to investigate the extent of the circulation of VIM‐1‐containing bacteria within gull populations in Southern France.

Our findings are all the more worrisome if we consider that gulls live in close contact with human populations since they feed on waste matter, notably in cities, and thus represent a bridge species for pathogens between wildlife and humans. Moreover, young yellow‐legged gulls can fly large distances from their native colony to their wintering sites that include the whole of the Rhone Valley and the French Atlantic coast (Sadoul & Pin, 2009). Thus, this species could favor the spread of carbapenem‐resistant bacteria, at least within France.

By contrast, we did not detect any carbapenem‐resistant *E. coli* in slender‐billed gulls. This species does not breed in the same colonies as YLG, but SBG and YLG can share resting sites and are thus frequently in contact. Furthermore, the two colonies we studied are located only 110 km apart. This distance can easily be covered by a gull within a day, meaning that these sites are not isolated from one another in terms of potential bacteria exchanges between the species we studied. As stated above, the two species differ by their diet, which suggests that the carriage of carbapenem‐resistant bacteria by YLG may be associated with their feeding habits and/or the habitat they visit to feed. In France, from January 2004 to March 2014, 913 infectious episodes associated with carbapenem‐resistant enterobacteria were reported, including 233 episodes due to *E. coli,* all of which were detected in hospitals (INVS 2014). Many of those episodes (481 out of 913) were linked to a previous stay of the patient in a foreign country (Crémet et al., 2012, Cuzon, Naas, Lesenne, Benhamou, & Nordmann, 2010; INVS 2014), which underscores the rarity of carbapenem‐resistant infection originating in France. Furthermore, the resistance gene we detected (*bla*_VIM‐1_) is uncommon in France, where it caused only 5% of the reported infections due to carbapenem‐resistant enterobacteria, the OXA‐48‐ and OXA‐48‐like genes being the most frequent in the country (74%) (INVS 2014). *bla*_VIM‐1_ is an integron‐borne metallo‐β‐lactamase gene which was first reported in *Pseudomonas aeruginosa* in Italy in 1996 (Lauretti et al., 1999). It encodes for a class B carbapenemase which also hydrolyzes all β‐lactams except monobactams, and evades all β‐lactamase inhibitors. VIM‐1‐bearing bacteria have been reported from clinical samples in Greece although they are beginning to spread in southwestern Europe, notably in Spain and Italy, while France seems, for now, to be less affected (Canton et al., 2012; Mathlouthi et al., 2016).

The phylogenetic analyses performed using phylotyping and three types of genetic markers (SNP, MLST and VNTR) clearly showed that yellow‐legged gulls, slender‐billed gulls, and humans share the same pool of *E. coli* strains. Our results confirm that *E. coli* exchanges are frequent between gulls and humans, as was previously demonstrated in the region (Bonnedahl et al., 2009). The occurrence of such exchanges highlights the potential risk of resistance spreading from gulls to humans (Stedt et al., 2014).

VIM‐1‐containing *E. coli* are closely related to carbapenem‐susceptible strains isolated from the two gull species and humans. Nevertheless, their group can be distinguished from the susceptible group through two genetic traits. First, PCR phylotyping showed that the 92 strains we studied included bacteria belonging to 8 phylogroups. No phylogroup was significantly more present than others in susceptible strains. By contrast, phylogroup A, to which some susceptible strains also belong, represented 81.8% of the VIM‐1‐bearing *E. coli*. The association between some phylogroups and antimicrobial resistance patterns is for now poorly understood. Nevertheless, several studies have already highlighted that phylogroup A *E. coli* are over‐represented within resistant strains isolated in France (Smati et al., 2013), including chromosomal AmpC β‐lactamase overproducers carried by humans (Corvec et al., 2007) as well as ESBL *E. coli* detected in cattle (Valat et al., 2012) and ampicillin‐resistant isolates from pigs (Bibbal, Dupouy, Prère, Toutain, & Bousquet‐Mélou, 2009). Further studies are still needed to determine if *E. coli* belonging to phylogroup A are more likely to acquire antimicrobial resistances and why. Carbapenem‐resistant strains tended to be less diverse than susceptible ones according to VNTR and SNP analysis. This lower diversity is consistent with the higher selection pressure, potentially linked with antimicrobial molecule presence, resulting in strong bottlenecks that are expected to have contributed to the emergence of resistant strains. This suggests that the resistance was recently acquired by the bacteria we isolated or that a selection pressure favored the expansion of a preexistent clone.

We report here the second isolation worldwide of carbapenem‐resistant enterobacteria from wild birds and the first detection in gulls. In‐depth molecular analysis of blaVIM genetic surroundings and features will be necessary in the future to fully understand the downstream impacts of our findings. Yet, here our aim was not to fully characterize the genetic background of carbapenem‐resistant *E. coli* carried by wildlife but rather to warn over the potential role of wild birds as carbapenem‐resistant bacteria carriers and spreaders. Our results are alarming enough to justify an urgent call for further studies. They also make it a matter of urgency to determine the contamination source of the bacteria we identified. The next step could be to extend our work to several other YLG and SBG colonies, including some in which the two species are breeding in neighboring islets, as well as to repeat sampling other time to improve our knowledge of VIM‐1 carrying bacteria circulation in French gull populations. Such extended sampling could also help in disentangling the respective roles of feeding habits and environmental pollution in carbapenem‐resistant bacteria contamination. To complete this analysis, it would also be necessary to search for resistant bacteria within the environment, especially in water, since it has previously been shown that rivers and coastal areas can contain carbapenem‐resistant bacteria even when those pathogens are rare in the surrounding human populations (Aubron, Poirel, Ash, & Nordmann, 2005; Montezzi et al., 2015). More generally, our work highlights the urgent need to develop more bridges between studies focusing on wildlife and humans in order to improve our knowledge of resistant bacteria dynamics. Such bridges will be a key factor in enabling us to efficiently face the challenge of antimicrobial resistance in the future.

## Data Accessibility

Molecular data will be archived in the following database: http://enterobase.warwick.ac.uk/.

## Conflict of Interest

None declared.
