# Supplementary material for: VIM‐1 carbapenemase‐producing Escherichia coli in gulls from southern France
Source: Ecol Evol. 2017 Jan 25;7(4):1224–32. doi: 10.1002/ece3.2707 (PMC5305998; doi:10.1002/ece3.2707)

| **Supplementary Information – Table S1.** SNP profiles from *Escherichia coli* isolates. SNP from mdh (450) was not shown because the difference of SNP of this gene was considered in MLST sequencing. SNP studied were from Sheludchenko et al., 2010. | | | | | | | |
| --- | --- | --- | --- | --- | --- | --- | --- |
| Isolate | fadD (234) | clpX (267) | uidA (138) | clpX (177) | clpx (234) | lysP (198) | icdA (177) |
| Ec001 | C | C | C | G | C | C | T |
| Ec003 | C | C | C | G | C | C | T |
| Ec004 | C | C | C | G | C | C | T |
| Ec005 | C | C | C | G | C | C | T |
| Ec006 | C | C | C | G | C | C | T |
| Ec007 | C | C | C | G | C | C | T |
| Ec008 | C | C | C | G | C | C | T |
| Ec010 | C | C | C | G | C | C | T |
| Ec011 | C | C | C | G | C | C | T |
| Ec012 | C | C | C | G | C | C | T |
| Ec013 | T | C | T | G | T | C | C |
| Ec014 | A | C | C | G | C | C | T |
| Ec015 | C | C | C | G | C | C | T |
| Ec016 | C | C | C | G | C | C | T |
| Ec017 | A | C | C | G | C | C | T |
| Ec019 | C | C | C | G | C | C | T |
| Ec020 | C | C | C | G | C | C | T |
| Ec021 | C | C | C | G | C | C | T |
| Ec022 | T | C | T | G | T | C | C |
| Ec023 | C | C | C | G | C | C | T |
| Ec024 | C | C | C | G | C | C | T |
| Ec025 | C | C | C | G | C | C | T |
| Ec026 refA | C | T | C | A | C | C | T |
| Ec027 refB1 | A | T | C | G | C | C | T |
| Ec028 refB2 | T | C | C | G | T | T | C |
| Ec029 refD | T | C | T | G | C | C | C |
| Ec030 | A | C | C | G | C | C | C |
| Ec031 | A | T | C | G | C | C | T |
| Ec032 | C | T | C | G | C | C | T |
| Ec033 | A | C | C | G | C | C | C |
| Ec035 | C | C | T | G | C | C | C |
| Ec037 | C | C | C | A | C | C | T |
| Ec038 | T | C | T | A | C | C | C |
| Ec039 | C | C | C | A | C | C | T |
| Ec040 | C | T | C | G | C | C | T |
| Ec042 | C | T | C | A | C | C | C |
| Ec043 | C | C | T | G | C | C | C |
| Ec044 | C | C | C | G | C | C | T |
| Ec045 | A | C | C | G | C | C | C |
| Ec046 | A | C | C | G | C | C | C |
| Ec047 | A | T | C | A | C | C | C |
| Ec048 | A | T | C | G | C | C | T |
| Ec049 | A | T | C | G | C | C | T |
| Ec050 | T | C | T | G | C | C | C |
| Supplementary Information - Table 3 (continue). | | | | | | | |
| Isolate | fadD (234) | clpX (267) | uidA (138) | clpX (177) | clpx (234) | lysP (198) | icdA (177) |
| Ec051 | C | T | C | G | C | C | C |
| Ec052 | C | C | C | G | C | T | T |
| Ec053 | A | T | T | G | C | C | T |
| Ec054 | T | C | T | G | C | C | C |
| Ec055 | T | C | C | G | C | C | C |
| Ec056 | C | T | C | G | C | C | T |
| Ec057 | A | C | T | G | C | C | T |
| Ec058 | A | C | C | G | C | C | T |
| Ec059 | T | C | C | G | C | C | C |
| Ec061 | C | T | C | G | C | C | T |
| Ec062 | A | C | C | G | C | C | T |
| Ec063 | C | T | C | G | C | C | T |
| Ec064 | C | C | C | G | C | C | T |
| Ec065 | C | C | C | G | C | C | T |
| Ec066 | T | C | T | G | C | C | C |
| Ec067 | A | C | C | G | C | C | T |
| Ec068 | C | C | C | G | C | C | T |
| Ec069 | C | C | C | G | C | C | T |
| Ec070 | A | T | T | A | C | C | T |
| Ec071 | C | C | C | G | C | C | T |
| Ec072 | C | T | C | G | C | C | T |
| Ec073 | T | C | T | G | C | C | C |
| Ec074 | A | T | C | G | C | C | T |
| Ec075 | A | C | T | G | C | C | C |
| Ec076 | A | T | T | A | C | C | C |
| Ec077 | C | C | C | G | C | C | T |
| Ec078 | C | C | C | G | C | C | T |
| Ec079 | T | C | T | A | C | C | C |
| Ec080 | T | C | T | G | C | C | C |
| Ec081 | T | C | C | G | C | T | C |
| Ec082 | A | T | T | G | C | C | C |
| Ec083 | A | T | C | A | C | C | T |
| Ec084 | C | T | C | G | C | C | T |
| Ec085 | C | C | C | G | C | C | T |
| Ec086 | T | C | C | G | C | T | C |
| Ec087 | A | T | T | A | C | C | C |
| Ec088 | T | C | C | G | T | T | C |
| Ec089 | C | C | C | G | C | C | T |
| Ec090 | T | C | C | G | C | T | C |
| Ec091 | T | C | C | G | C | T | C |
| Ec092 | T | C | C | G | C | T | C |
| Ec093 | C | C | C | G | C | C | T |
| Ec094 | T | C | T | A | C | C | C |
| Ec095 | T | C | T | A | C | C | C |
| Ec096 | C | C | C | A | C | C | T |
| Ec097 | T | C | C | G | C | C | C |
| Ec098 | A | T | C | A | C | T | T |
| Ec099 | C | C | T | A | C | C | C |

**Supplementary Information - Figure S1. Minimum spanning tree of 79 of the studied *Escherichia coli* strains based on MLST and SNPs.** The 13 strains for which part of the VNTR data was missing were excluded. The phylogroups are shown as ovals. Clonal complexes are indicated by symbols proportional in size to the number of strains within them. Black lines connecting strains indicate that they differ by (i) one MLST gene (bold thick lines), (ii) seven markers (five MLST genes and two SNPs (the thinnest lines)).


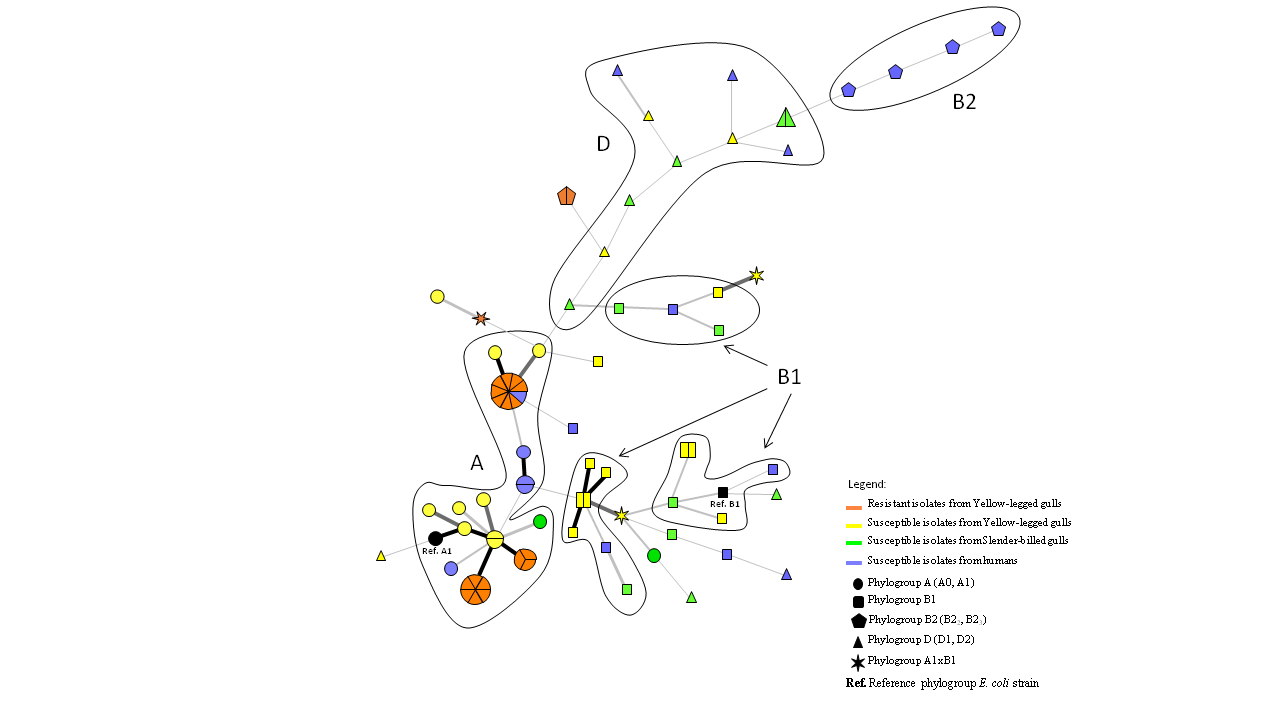

Supplement: Supplementary file 1 [file ECE3-7-1224-s001.docx]
